# Cognitive-Attentional Syndrome Moderates the Relationship Between Fear of Coronavirus and Symptoms of Coronavirus-Specific Health Anxiety

**DOI:** 10.1007/s41811-022-00147-9

**Published:** 2022-10-25

**Authors:** Joachim Kowalski, Łukasz Gawęda

**Affiliations:** Experimental Psychopathology Lab, Institute of Psychology, Polish Academy of Sciences, Jaracza 1, 00-378 Warsaw, Poland

**Keywords:** Metacognitive therapy, Hypochondriasis, Fear, Anxiety, Cognitive-attentional syndrome, Adjustment reaction

## Abstract

This study was aimed at exploring the possible roles of the cognitive attentional syndrome (CAS) and metacognitive beliefs in moderating the relationships between fear of coronavirus during the pandemic and health anxiety. Because some symptoms of health anxiety may overlap with symptoms of other anxiety disorders, we also tried to ascertain whether our hypothesized relations would be maintained when taking other anxiety disorder symptoms into account. We hypothesized that CAS strategies and meta-beliefs would play a role in the progression from fears of the coronavirus to coronavirus health anxiety. The method done was a cross-sectional study with *n* = 783 participants who completed questionnaires on fear of coronavirus, coronavirus-specific health anxiety, CAS, and symptoms of anxiety disorders. Fear of coronavirus and coronavirus health anxiety are correlated with medium effect size. CAS and metacognitive beliefs moderate the relationship between fear of coronavirus and symptoms of coronavirus-specific health anxiety. CAS predicts a unique part of health anxiety symptoms variance above symptoms of other anxiety disorders. The results of this cross-sectional study preclude causal inferences but tentatively suggest that CAS strategies may play a role in moderating the relationship between fear of coronavirus and coronavirus-related health anxiety. These relationships were obtained after controlling for variance shared with agoraphobia, social phobia, and general physical symptoms of anxiety.

## Introduction

Feeling fear or anxiety may be viewed as a part of an adjustment reaction to the threatening situation. The pandemic of SARS-CoV-2, or coronavirus, is widely regarded as such a situation. A higher level of anxiety in reaction to the current situation is observed population-wide (Mertens et al., 2020; Özdin & Bayrak Özdin, 2020; Rajkumar, 2020; Tull et al., 2020). Some people may experience an exacerbation of their pre-existing health anxiety symptoms (Asmundson et al., 2020) or start to experience symptoms of health anxiety in relation to the pandemic situation (Asmundson & Taylor, 2020). Recent data show a positive relationship between anxiety associated with coronavirus and trait health anxiety during the pandemic (Jungmann & Witthöft, 2020).

People with elevated health anxiety of coronavirus may engage in maladaptive coping behaviours. Such behaviours repeatedly preoccupy healthcare workers, seeking unnecessary medical attention and reassurances or avoiding the healthcare system altogether (Asmundson & Taylor, 2020). A better understanding of mechanisms that underlie health anxiety during the current pandemic may improve diagnostic and therapy protocols. Among others, a metacognitive approach to psychopathology, namely the Self-Regulatory Executive Function (S-REF) model (Wells, 2019; Wells & Matthews, 1996), is one of the approaches that offer insight into the interplay between fear and health anxiety.

The metacognitive approach to psychopathology points to the cognitive-attentional syndrome (CAS) as a factor underlying the transformation of common negative cognitive and emotional phenomena into lasting emotional discomfort and disorders (Kowalski & Dragan, 2019; Wells, 2009, 2019). CAS is a set of cognitive and behavioural strategies originating from maladaptive metacognitive beliefs. In this approach, health anxiety may be conceptualized as a product of maladaptive metacognitive beliefs and associated CAS strategies. These strategies are rumination, worry, threat monitoring, suppressing thoughts and emotions, and asking for reassurance (Wells, 2009). For instance, a person with tendencies for health anxiety may exhibit positive metacognitive beliefs about CAS strategies, like “checking for symptoms will keep me safe” and engage in CAS in the form of, e.g. worrying about the possibility of being ill, concentrating on bodily functions and repeatedly checking for breathing difficulties. This will eventually lead to a perception of breathing problems, more worry, and activation of negative metacognitive beliefs—e.g. about the dangerousness of thoughts and their uncontrollability, like “worrying like this can make me sick”—and further preoccupation with CAS strategies (Bailey & Wells, 2013; Wells, 2009). In other words, in this approach, maladaptive metacognitive beliefs resulting from them CAS are responsible for the transformation of common and transient anxiety into symptoms of health anxiety disorder. Previous studies confirm the role of maladaptive metacognitive beliefs and CAS strategies in health anxiety symptomatology (Bailey & Wells, 2013, 2015a; Kaur et al., 2011; Melli et al., 2018). There is also preliminary evidence that metacognitive therapy may effectively reduce health anxiety symptoms, CAS, and metacognitive beliefs alike (Bailey & Wells, 2014).

In the current study, we examined the relationships between anxiety related to coronavirus and metacognitive factors, i.e. CAS and metacognitive beliefs, and how symptoms of cognitive-attentional syndrome modulate relationships between fear related to coronavirus and symptoms of coronavirus-specific health anxiety. We hypothesized that metacognitive factors would be positively associated with fear related to coronavirus and coronavirus health anxiety. We hypothesized that CAS strategies and metacognitive beliefs would moderate this relationship. With higher CAS symptoms, the relationship between fear of coronavirus and coronavirus-specific health anxiety will be stronger.

Furthermore, we acknowledge that on a self-assessment level, symptoms of health anxiety may be observed as a result of less specific anxiety disorder symptoms, i.e. that general levels of anxiety may be a factor that predicts the level of health anxiety symptoms. Because of that, it was warranted to examine whether symptoms of CAS and its interaction with fear of coronavirus can uniquely predict levels of coronavirus-specific health anxiety when taking other, less specific anxiety symptoms into account. We hypothesized that elements of CAS and interactions of CAS and fear related to coronavirus would predict levels of coronavirus-specific health anxiety over and above fear related to coronavirus and other types of anxiety disorders symptoms that is agoraphobic, social phobia, and vegetative symptoms.

## Methods

### Procedure and Sample Selection

The study was conducted between 21st and 28th April 2020, during the COVID-19 pandemic lockdown. Details on the socio-legal context are provided in our previous work (Kowalski et al., 2020). The study was conducted online, using LimeSurvey software. We recruited participants via social media advertisements. Users were asked to participate in the study and pass it on to their friends, so a mixture of convenience and snowball sampling was used. Participation in the study was voluntary, and participants gave their informed consent. The study was approved by the Research Ethics Committee at the Institute of Psychology, Polish Academy of Science (motion number 13/IV/2020).

Eight hundred forty participants completed all surveys. As the lack of presence of a particular affliction is a diagnostic criterium for health anxiety (American Psychiatric Association, 2013), we asked participants about going through an upper respiratory tract infection in the last week. To reliably assess health anxiety connected to COVID-19, we excluded all participants who gave an affirmative answer to the item “I was going through an infection – at least a few days of cough, fever and fatigue”. Those *n* = 57 participants were excluded, leaving *n* = 783 participants that were considered in the final analyses. The demographic characteristics of this sample were as follows: 73.1% of participants were female, 26.1% were male, and 0.9% declared other gender or did not want to disclose one. The participants were 18 to 77 years old, with a mean of 29.77 (± 10.27). Five percent of participants had primary or vocational education, 35.9% had secondary education, and 59.1% had higher education. 16.1% of participants lived in the country, 27.3% in a town below 100 k inhabitants, 22% in a small city between 100 and 500 k inhabitants, and 34.6% in a large city above 500 k inhabitants.

### Measures

- *Fear related to coronavirus*—measure consisting of 5 items assessed on a scale from 1 to 7 related to the anxiety of (1) contracting coronavirus, (2) family member contracting coronavirus, (3) infecting other people with coronavirus, (4) someone infecting you with coronavirus, and (5) worsening of the financial situation due to coronavirus. Higher scores indicate greater levels of anxiety associated with coronavirus. This measure had a satisfactory internal consistency of Cronbach’s *α* = 0.81.
- *Coronavirus-specific health anxiety*—measure consisting of 9 items assessed on a scale from 1 to 7 related to symptoms of health anxiety in the context of coronavirus and one additional item controlling for the presence of infection (as described in the “Procedure and Sample Selection” section). It was based on diagnostic criteria of Illness Anxiety Disorder from DSM-5 (American Psychiatric Association, 2013), Hypochondriasis from ICD-10 (World Health Organization, 1993), and descriptions from the therapy protocol of health anxiety (Wells, 1997). The items were as follows: (1) I cannot stop worrying about contracting coronavirus, (2) I feel a need to repeatedly ask for reassurance if I’m safe from catching coronavirus, (3) Even slight mention of coronavirus makes me anxious, (4) I very often check my symptoms, i.e. touch lymph nodes or measure body temperature, (5) I seek medical assistance when there is the slightest possibility of something being wrong with me, (6) It was pointed out to me before, that my preoccupation with coronavirus is excessive, (7) I wouldn’t go to a hospital even if I had symptoms of coronavirus infection, (8) I try very hard not to think about contracting coronavirus, (9) I used to have similar concerns about other illnesses than coronavirus. The main nine items had a satisfactory internal consistency of Cronbach’s *α* = 0.82.
- *Cognitive-attentional syndrome questionnaire (CAS-1)*—is a short tool for assessing cognitive-attentional syndrome (Wells, 2009). In this study, we used a polish translation with a 2-factor solution (Kowalski & Dragan, 2019). It consists of 8 items relating to CAS strategies (i.e. worry, rumination, attention to threat, suppressing thoughts) and eight items relating to metacognitive beliefs. Items relating to metacognitive beliefs are originally assessed on a 0–100 scale, but for the study, answers were recoded to range from 0 to 8, as in other items. In the current study, strategies and beliefs subscales had acceptable internal consistencies of Cronbach’s *α* = 0.84 and *α* = 0.68, respectively.
- *Symptoms Checklist-27-plus (SCL-27-plus)*—is a comprehensive screening measure of different types of emotional disorders symptoms and pain (Hardt, 2008; Kuncewicz et al., 2014). It consists of five subscales measuring pain, depressive, agoraphobic, sociophobic, and vegetative symptoms. The whole questionnaire had an excellent *α* of 0.93. All subscales had satisfactory internal consistency ranging from *α* = 0.76 (pain subscale) to *α* = 0.93 (depressive symptoms subscale).

### Data Analysis Strategy

Firstly, we performed correlational analyses with Spearmans’s rho ranked correlation coefficient of all variables of interest. We subjected these analyses to a Bonferroni correction to account for the number of comparisons. Then, we tested hypotheses for CAS strategies and metacognitive beliefs moderating relationships between fear related to coronavirus and coronavirus-specific health anxiety symptoms using the Process macro (Hayes, 2012), model 1. Finally, we created hierarchical series of linear regression models predicting coronavirus-specific health anxiety symptoms, with vegetative, agoraphobic, and sociophobic anxiety symptoms, fear related to coronavirus, CAS-related variables, and interaction terms from moderation analyses as predictor variables.

Additionally, we performed confirmatory factor analysis in AMOS v27 for our two newly developed measures—fear related to coronavirus and coronavirus-specific health anxiety—as initial confirmation of construct validity. We used CFA criteria from a previously published study published in the ICOT (Nordahl & Wells, 2019).

## Results

Confirmatory factor analyses deemed that intended one-factor solutions for our newly developed measures are a good fit to the data. Fear related to coronavirus had a *χ*^2^ = 19.49, *p* < 0.001, CFI = 0.99, TLI = 0.97, RMSEA = 0.084, and SMRM = 0.033. Coronavirus-specific health anxiety had *χ*^2^ = 137.26, *p* < 0.001, CFI = 0.96, TLI = 0.94, RMSEA = 0.074, and SMRM = 0.039.

Correlational analyses revealed positive associations between all variables. Table 1 presents these analyses’ details and the variables’ mean values.

**Table 1:** Correlation coefficients between variables of interest

		1	2	3	4	5	6	m (sd)
1. Coronavirus-specific health anxiety								18.70 (9.06)
2. Fear related to coronavirus		0.47**						23.15 (6.31)
CAS-1	3. Strategies	0.35**	0.25**					24.55 (13.90)
	4. Beliefs	0.24**	0.12*	0.58**				32.88 (11.15)
SCL-27-plus	5. Vegetative	0.32**	0.22**	0.43**	0.33**			3.65 (3.96)
	6. Sociophobic	0.33**	0.18**	0.56**	0.47**	0.54**		4.82 (5.20)
	7. Agoraphobic	0.43**	0.37**	0.40**	0.34**	0.45**	0.53**	2.70 (3.81)

*CAS-1* cognitive-attentional syndrome questionnaire, *SCL-27-plus* Symptoms Checklist 27 plus. Note: Significance values were subjected to a Bonferroni correction accounting for number of comparisons (21). *p < 0.01, **p < 0.001

To test moderation hypotheses, we ran two analyses with CAS strategies and metacognitive beliefs as moderator variables. In both models, the predictor was coronavirus-related fear and predicted variable was symptoms of coronavirus-specific anxiety. Both models and respective interaction terms were significant. Model with CAS strategies—*F*(3,779) = 121.52, *p* < 0.0001, *R*^2^ = 0.32—revealed significant moderating effect of CAS strategies on fear related to coronavirus predicting symptoms of coronavirus-specific anxiety, *b* = 0.019, *se b* = 0.003, *p* < 0.0001. Model with metacognitive beliefs—*F*(3,779) = 98.30, *p* < 0.0001, *R*^2^ = 0.27—revealed significant moderating effect of metacognitive beliefs on fear related to coronavirus predicting symptoms of coronavirus-specific anxiety, *b* = 0.018, *se b* = 0.004, *p* < 0.0001.

**Fig. 1:**
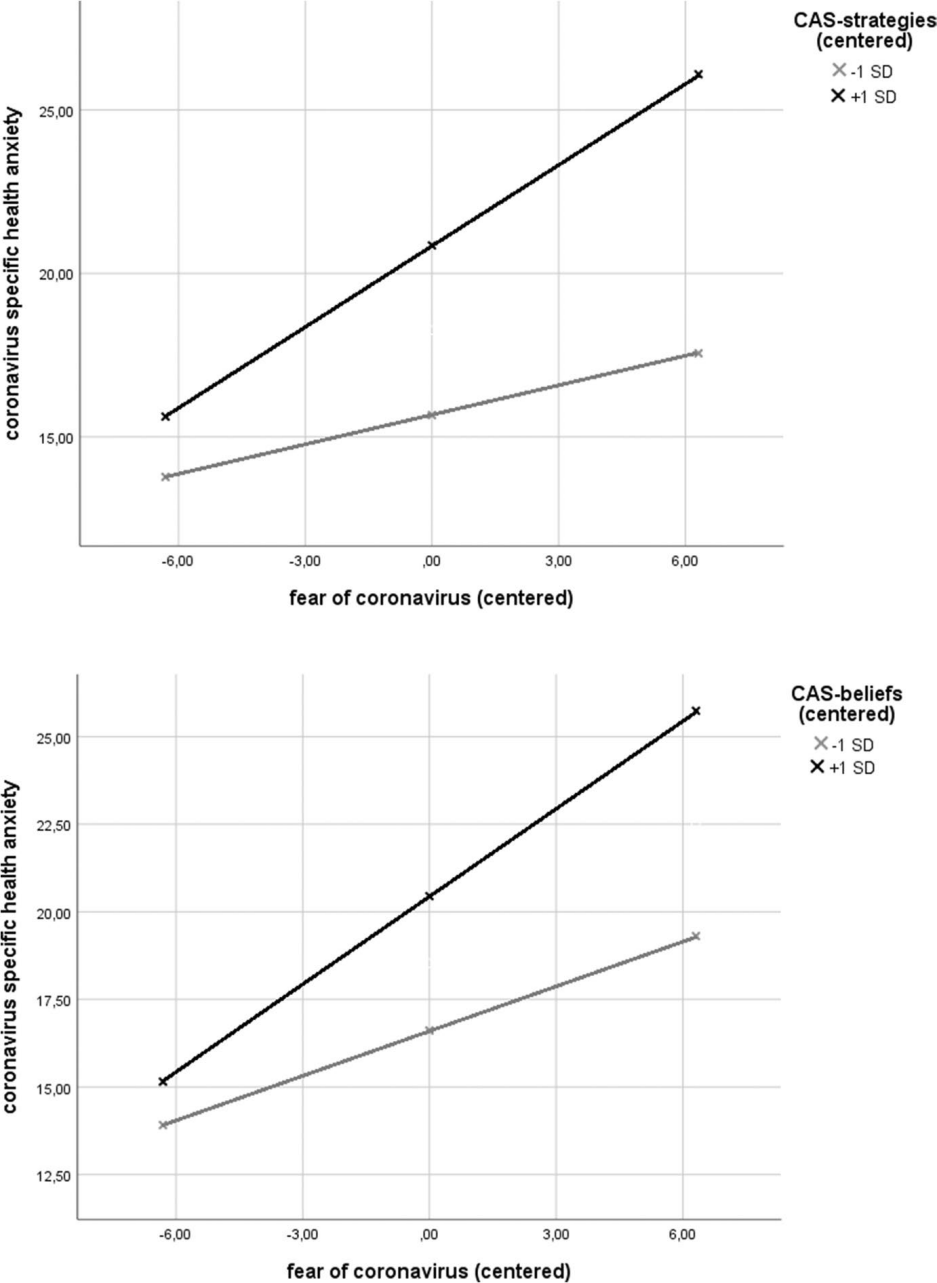
Moderating effects of CAS strategies and CAS beliefs on the relationship between fear related to coronavirus and symptoms of coronavirus-specific health anxiety

Simple slopes analysis revealed that CAS strategies predict effects of coronavirus-related fear on coronavirus-specific health anxiety on either low (− 1 sd, *b* = 0.30, *se b* = 0.06, *p* < 0.0001) and high levels (+ 1 sd, *b* = 0.83, *se b* = 0.06, *p* < 0.0001), with most prominent effect at high levels of CAS strategies. Similarly, effects for metacognitive beliefs showed low (− 1 sd, *b* = 0.43, *se b* = 0.06, *p* < 0.0001) and high levels (+ 1 sd, *b* = 0.84, *se b* = 0.06, *p* < 0.0001) of these beliefs predicting effect of coronavirus-related fear on coronavirus-specific health anxiety, with most prominent effect at high levels of metacognitive beliefs. Figure 1 shows details of these analyses.

Finally, we created a series of regression models with all variables of interest and interaction terms. Table 2 shows details of the models.

**Table 2:** Regression models of variables and interaction terms predicting coronavirus-specific symptoms of health anxiety

Step	Variable name	*F* change	Δ*R*^2^	*β*	*t*
1		108.22	0.35**		
	Coronavirus-related fear			0.32	10.30**
	SCL vegetative			0.12	3.39**
	SCL sociophobic			0.05	1.37
	SCL agoraphobic			0.30	7.74**
2		10.00	0.02**		
	Coronavirus-related fear			0.29	9.45**
	SCL vegetative			0.10	2.80*
	SCL sociophobic			− 0.02	− 0.47
	SCL agoraphobic			0.29	7.73**
	CAS-1 strategies			0.14	3.63**
	CAS-1 beliefs			0.03	0.81
3		15.70	0.02**		
	Coronavirus-related fear			0.32	10.23**
	SCL vegetative			0.10	2.70*
	SCL sociophobic			0.00	0.09
	SCL agoraphobic			0.26	6.74*
	CAS-1 strategies			0.15	3.88*
	CAS-1 beliefs			0.03	0.98
	Coronavirus-related fear X CAS-strategies			0.13	3.85**
	Coronavirus-related fear X CAS-beliefs			0.04	1.26

*CAS-1* cognitive-attentional syndrome questionnaire, *SCL* Symptoms Checklist 27 plus. *p < 0.01, **p < 0.001

## Discussion

The current study examined the role of CAS symptoms in the relationship between fear related to coronavirus and coronavirus-specific health anxiety, controlling for generic anxiety symptoms in a non-clinical sample. In general, obtained results are in line with the hypotheses. We demonstrated that CAS strategies and metacognitive beliefs moderate the relationship between fear related to coronavirus and coronavirus-specific health anxiety symptoms. In the regression model, only CAS strategies and respective interaction term predicted variance of coronavirus health anxiety over and above symptoms of other anxiety disorders; however, metacognitive beliefs did not.

Correlational analyses between variables showed positive and moderate associations. Unsurprisingly, variables related to anxiety and feeling of fear related to each other. Also, CAS strategies and metacognitive beliefs were associated with other anxiety-related variables, e.g. symptoms of social phobia, agoraphobia, and vegetative symptoms. Notably, symptoms of coronavirus-specific health anxiety show more robust relationships with CAS strategies, metacognitive beliefs, and symptoms of anxiety disorders than fear related to coronavirus. This points out the plausibility of the assumed difference between coronavirus-related fear and coronavirus-specific health anxiety. The obtained results agree with a previous study showing positive relationships between general anxiety, health anxiety, and maladaptive metacognitive beliefs (Melli et al., 2018).

The present study was concerned with factors that influence the development of coronavirus-specific health anxiety from initial fears of the virus. Although fear of coronavirus is a natural and adaptive response, health anxiety symptoms are not as they involve an extensive preoccupation with the possibility of contracting the illness. Specifically, the main goal of this cross-sectional study was to test whether CAS strategies and metacognitive beliefs specified in Wells’ (2009) metacognitive model might moderate or influence the association between fear of coronaviruses and symptoms of health anxiety particular to coronaviruses. With that, the study also examined whether elements of CAS predict a distinct portion of the variance in coronavirus-specific health anxiety symptoms after adjusting for the effects of more general anxiety symptoms. The results of this study provide support for the difference between coronavirus fear and coronavirus-related health anxiety. In addition, they generally offer some support for the predictions based on Wells’ metacognitive model (Bailey & Wells, 2013, 2015a, 2016a, b).

The results indicated that both CAS strategies and metacognitive beliefs moderate the relationship between fear related to coronavirus and coronavirus-specific health anxiety symptoms. When CAS strategies and metacognitive beliefs were entered into the same regression model, only the CAS strategies and their interaction with coronavirus fear predicted a unique portion of variance in coronavirus health anxiety, over and beyond other anxiety disorder symptoms, but metacognitive beliefs did not. After adjusting for the effects of symptoms of agoraphobia, social phobia, and vegetative anxiety, only CAS strategies and their related interaction terms predicted variance in coronavirus-specific health anxiety. However, we were unable to detect the impact of metacognitive beliefs, perhaps due to a shared variation with different types of anxiety symptoms (Wells, 2007; Wells & Carter, 2001).

The results confirmed that, in contrast to fear associated with coronavirus, symptoms of coronavirus-specific health anxiety have more robust associations with CAS strategies, metacognitive beliefs, and symptoms of anxiety disorders. These results support the validity of separating coronavirus-specific health anxiety from coronavirus-related worry. They also fit with a prior study that found links between general anxiety, health anxiety, and unhelpful metacognitive beliefs (Melli et al., 2018).

Overall, our findings support adapting the S-REF model to the context of health anxiety related to COVID-19. The results suggest that CAS is important in the relationship between fear of coronavirus and coronavirus-related health anxiety, so they may have clinical implications. Both CAS and maladaptive metacognitive beliefs are a central tenet of the metacognitive approach to psychotherapy (Wells, 2009, 2019, Kowalski et al., 2019). At the same time, some of the CAS strategies, like rumination and worry, are the target of various psychological approaches, like rumination-focused CBT, mindfulness-based approaches (Watkins, 2015), or CBT protocols for generalized anxiety disorder (Covin et al., 2008). So, targeting CAS may be a way to diminish the relationship between fear of illness and self-assessed symptoms of health anxiety disorder.

Our study has several limitations. Data were collected online with a mixture of convenience and snowball sampling methods, which resulted in our sample not corresponding to the general population’s characteristics and thus limiting our results’ generalizability. The second limitation is that we used a community sample, not a clinical one. Nevertheless, it may be further hypothesized that the observed interplay between variables of interest may be amplified in clinical populations, and our results can be translated into therapeutic practice. However, firm conclusions are not eligible at the current state of research. Another limitation is that our data is cross-sectional and correlational. We had no basis for causal or temporal-precedence inferences. Future studies should use experimental procedures to demonstrate the role of CAS in developing the common experience of anxiety or fear into symptoms of hypochondriasis. There is also a possibility to use measures of metacognition concentrating more directly on health anxiety (Bailey & Wells, 2015b). Future studies can also seek relationships between actual physical complaints coinciding with specific symptomatology, health anxiety, and CAS. We could not do so, as we had to exclude participants that declared going through an infection right before or during the study. Another direction for future studies would be employing clinician-administered measures of health anxiety.

To sum up, we have found preliminary evidence that CAS strategies and metacognitive beliefs moderate the relationship between fear of coronavirus and symptoms of coronavirus-specific health anxiety. Higher levels of CAS indicate stronger relationships between those two variables of interest. We demonstrated that CAS strategies predict a unique part of health anxiety symptoms variance, over and above symptoms of social phobia, agoraphobia, and vegetative symptoms. We suggest the usefulness of screening for CAS in different healthcare settings. Taken together, our results suggest that CAS and metacognitive beliefs may play a role in the development of an adaptive response, like fear of coronavirus, into symptoms of health anxiety associated with this particular illness.
